# The Relationship Between Work During College and Post College Earnings

**DOI:** 10.3389/fsoc.2019.00078

**Published:** 2019-12-10

**Authors:** Daniel Douglas, Paul Attewell

**Affiliations:** ^1^Trinity College, Hartford, CT, United States; ^2^The Graduate Center, City University of New York, New York, NY, United States

**Keywords:** postsecondary education, undergraduate students, degree completion, work, earnings

## Abstract

Prior research suggests that undergraduates employed during term time are less likely to graduate. Using transcript data from a large multi-campus university in the United States, combined with student earnings data from state administrative records, the authors find that traditional-age students who worked for pay during college on average earned more after leaving college than similar students who did not work. This post-college earnings premium is on par with the benefit from completing a degree, even after controlling for demographic and academic achievement characteristics, across various student sub-groups, and including models that account for selection bias. Implications of these findings for theories of education and social stratification, and for educational policy are considered.

The majority of today's undergraduates (62%) work for pay while enrolled in college (Carnevale et al., 2015). A large research literature (summarized below) has focused on the short-term consequences of working during college—on grades, number of credits taken, and graduation. Such studies have predominantly reported negative effects from student employment (Neyt et al., 2017). This paper argues that prior research has largely overlooked an important aspect of the working student phenomenon. Examining a longer period and focusing on *earnings after college* reveals a substantial positive aspect of student employment during college.

Analyses presented below indicate that undergraduates from a public university system who worked for pay during college had substantially higher earnings years later after, compared to counterparts who were not employed while enrolled. This long-term economic benefit associated with paid employment while in college held for women and for men; for racial/ethnic minorities; for community college as well as 4-year college entrants; for those who had no work experience before starting college; and most notably even among those who did not complete a degree.

A substantial post-college earnings premium associated with working during college was observed in models that addressed selection effects, as well as in conventional regression analyses. Moreover, this wage premium was not a reflection of college majors, nor of academic performance, since models that controlled for these covariates also showed the benefit. Finally, the earnings advantage was evident from immediately after leaving college until data collection ended 15 years later.

The post-college earnings premium associated with working during college was sizable. Even undergraduates who earned modest amounts while enrolled earned significantly more after graduation, a boost comparable to the earnings increase associated with completing a degree. Students who worked for pay throughout their college years experienced a much larger wage premium than those who worked for fewer semesters.

The analysis presented below suggests the need for a reconceptualization of the working student phenomenon that goes beyond degree completion focus of the literature. Earlier research portrayed working during college as a risky if sometimes unavoidable activity, at best a distraction from the process of completing a degree, and at worst a cause of dropping out. For this large sample of students from non-elite colleges, these findings suggest on the contrary that employers pay a wage premium for three things: completing a credential; accruing college credits (irrespective of completion); and a record of sustained work experience while in college. This non-elite context is important given that most students attend colleges at this level of selectivity in the US. Undergraduates who both work during college and complete a degree gain the most in terms of a post-college earnings advantage. That is the optimal outcome. However, college students who accumulate credits short of a degree while establishing a work history also benefit from higher pay after entering the labor market.

This paper first reviews the literature on the scope, benefits, and drawbacks of college student work during term time. It then discusses theories linking employment during college to post-college earnings. The analyses of employment and post college earnings follow, concluding with a discussion of the implications for theory and policy.

## Prior Research

### Who Works While in College?

The National Postsecondary Student Aid Study (NPSAS) is a nationally-representative study of undergraduate students. NPSAS' definition of working students focuses on employment during term-time outside the university, deliberately excluding campus-related jobs such as work-study and work during summer break (National Center for Education Statistics, 2015, p. 9). It therefore provides a conservative estimate of how undergraduate employment. We used the National Center for Education Statistics' (NCES) online analysis tool to obtain the estimates provided in Table 1 (For estimates from an earlier wave of NPSAS, see Perna et al., 2007).

**Table 1:** Undergraduate student work during term time.

**Characteristic**	**Proportion**
**Work intensity**	
Not working	38%
1–20 h per week	21%
More than 20 h per week	41%
The proportion of each of the following sub-groups who did any paid work during term time:	
**Gender**	
Female	63%
Male	61%
**Race/Ethnicity**	
Asian	49%
Black/African-American	59%
Hispanic	61%
White	65%
**Parent's annual income**	
Less than $30,000	54%
$30,000–$64,999	59%
$65,000–$149,999	60%
$150,000 or more	54%
**Institutional level**	
2 year colleges	64%
4 year colleges	60%
**Institutional selectivity (4 year colleges only)**	
Unselective/Open admissions	69%
Minimally selective	60%
Moderately selective	59%
Highly selective	52%

*Source. Authors calculations of the National Postsecondary Student Aid Survey (NPSAS)*.

According to the NPSAS, 62.3% of undergraduates were employed during term time. This rate did not vary greatly by gender, race, or parental income. Employment is more common at 2-year and less selective colleges, where many students attend part-time and are disproportionately from lower-income families. But even at highly-selective 4-year institutions, about half of the students work (Perna et al., 2007). Despite research like this suggesting that working undergraduates have been the norm for decades (Kena et al., 2016), a stereotype based on the non-working college student pervades popular culture and informs educational policy (Choy, 2002; Tuttle et al., 2005).

### Why Do Undergraduates Work?

NPSAS asked whether students could afford to attend college if they did not work: 54% of employed students answered “No.” Scholars have documented that many undergraduates face economic hardship while enrolled, including food and housing insecurity (Broton and Goldrick-Rab, 2016). St. John (2003); Goldrick-Rab (2016) and others have argued that current financial aid levels are inadequate for many undergraduates. Federal financial aid calculations include an estimate of “Expected Family Contribution” that researchers have shown many families cannot afford (Stringer et al., 1998; King, 2002; Goldrick-Rab, 2016).

Alongside the roughly half of working undergraduates who say they *have to work* are others who choose to work for less compelling reasons. Clydesdale's (2007) ethnography of freshmen finds that the academic side of college is a secondary priority compared to the development of practical life skills. Earnings can take on a symbolic meaning as a marker of adulthood. Beyond this, earnings have a practical function, paying for dating, entertainment and consumerism, averaging $1,000 a month in Clydesdale's study (2007, p. 111).

### Term Time Employment and Academic Performance

Evidence is mixed as to whether employment during term time helps or hinders students' academic performance during college. In one review, Riggert et al. (2006, p. 69) concluded that the empirical literature is “… marked by diversity and contradiction. Some studies suggested that student employment negatively affected academic performances, while others concluded that employment was neutral or even beneficial.” A more recent review by Neyt et al. (2017, p. 22) concludes that the associations between student employment on educational attainment are generally negative, and that more intensive work yielded worse academic outcomes.

The main mechanism advanced to explain negative effects of employment is a “time bind” that leads some working students into academic difficulties and higher rates of dropping out (Stinebrickner and Stinebrickner, 2003, 2004). Tinto (1993, p. 64) argues that full-time employment limits time for interaction with other students and faculty, leading to poor social integration and to higher rates of student drop-out. Astin (1993, p. 358) reports that colleges where many students work have lower sanctions against dropping out. Bozick (2007, p. 271–273) reports that working in moderation does not seem to impede students' academic progress, but noting that “working more than 20 h a week during the first year of college … limits students' ability to sustain enrollment.”

Studying the effects of student employment on academic outcomes remains an active field, especially among labor economists. Darolia (2014) reports “little discernible impact of working on students' grades … [however] increased work intensity results in fewer credits completed in each term by full-time students … This may contribute to increasing time-to-degree…” Triventi (2014) reports for Italy that working during college, especially intensive work, is negatively associated with academic progress. Scott-Clayton and Minaya (2016) examine campus work study, finding on average that work-study students experience better academic outcomes, but they note heterogeneity in effects such that for some subgroups work study is associated with worse academic outcomes.

This burgeoning literature focuses on the effects of working during college on academic progress *in the short run*. This paper represents a shift in focus away from college grades, retention and graduation toward a consideration of longer-term post-college outcomes—for which there is far less research, which we now review.

### Term Time Employment and Post-College Earnings

Pascarella and Terenzini (2005, p. 520) conclude that working during college helps secure employment after graduation but does not enhance later earnings. Mayhew et al. (2016) find very little available evidence on the association between work during college on subsequent earnings, but note that studies show a generally positive impact. Other studies do find that term-time employment is associated with higher earnings after graduation. Titus (2010) analyzed a sample of US undergraduates in the Beginning Postsecondary Students (BPS 96/01) longitudinal survey and found that the boost in post-college earnings associated with working during a student's third year of college was higher than the wage benefit from completing a degree. Molitor and Leigh (2005) documented a large effect (4 to 7 percent increase) of working during college on later earnings especially for male students in 2-year institutions. Similarly, Stephenson (1982) found significantly higher post-college wage rates for US males who worked during college in the National Longitudinal Survey of Youth (NLSY). Gee San (1986) reported similar findings, noting that effects on earnings appeared to be highest 3 years after completing college. However, using the same data, Ehrenberg and Sherman (1987) found no direct effect of working on post-college earnings. Similarly, Hotz et al. (2002) found payoffs to NLSY men who worked during college that became non-significant when analyzed using dynamic selection models. The data source utilized in this paper contains a far larger group of undergraduates than either the NLSY or the BPS 96/01, which facilitates analysis of various student subgroups; these data also contain comprehensive long-term earnings data from state administrative records, rather than student self-reports clustered shortly after college. International studies of work during college also point to higher post-college earnings for students who work while enrolled (Hakkinen, 2006; Jewell, 2014).

In sum, some prior research reports a significant association between working during college and having higher earnings after college, but the number of studies is not large. This paper will address the same research question: is work during college associated with higher post college earnings?—using administrative data that reports college and post-college earnings rather than the self-reported earnings data used in those earlier studies.

### Theory, Mechanisms, and Causation

An extensive body of theory considers the relationship between educational attainment, work experience, job assignment, and earnings (Bills, 2003). Most of these theories were formulated when the norm was first to complete one's education and then to enter the labor market, rather than the present context where most undergraduates work for pay while enrolled in college.

Human Capital theory posits that both formal education and work experience result in the accumulation of skills and knowledge that increase an individual's productivity and consequently are rewarded with higher pay. In Mincer's (1974) widely-used formulation, the log of earnings is considered an additive function of an individual's years of education plus work experience. For Mincer, the earnings return to education was viewed primarily as a “compensating difference.” Individuals who spend more years in education have foregone income they would have earned if instead they had entered the labor force. Higher wage rates for college-educated employees compensate for this.

Extensions of the Human Capital framework have modified Mincer's framework to allow for non-linear effects of the length of work experience and of years of education (Manski, 1989; Altonji, 1993; Aina et al., 2018). These include modeling the effects of stopping out of college—hypothesized by Fortin and Ragued (2017) to cause skill depreciation and obsolescence—and the effects of delayed time to degree on post-college earnings (Aina and Pastore, 2012). Economists have also broadened their concept of Human Capital beyond cognitive skills to include soft skills, character traits, and attitudes (Heckman and Kautz, 2012).

The Human Capital perspective implies that college students' term-time employment experiences will produce skills valued by future employers. There is a rich literature on work-based informal learning (for two reviews see Le Clus, 2011; Manuti et al., 2015). One implication is that extensive work experience during college, even in low-paying jobs, may produce competencies that are valued by employers and therefore result in higher post-college wages.

A second mechanism by which employment during college may lead to increased earnings involves students' resumés. Screening and Signaling theories (Arrow, 1973; Spence, 1974; Bills, 2003) suggest that employers lack direct knowledge of a job candidate's skills and capacities when hiring and therefore use “screens” to select candidates they deem more likely to become good employees. Similarly, job candidates seek to amass and display “signals” that they would be superior employees. These screens and signals include educational credentials (degrees), but a resumé listing extensive work experience can also signal a job applicant's promise. Holzer and Neumark's (1999) study of hiring found that employers look for applicants with more stable work histories. Similarly, a survey conducted by the *Chronicle of Higher Education* indicates that employers weigh prior work experiences (both paid work and internships) more heavily than indicators of educational achievement when hiring recent college graduates (Fischer, 2013).

A third perspective on the benefits of employment during college highlights the importance of references and social networks for gaining post-college jobs. Granovetter (1995) documented the importance of networks in providing information about job openings or for recommending a person in one's social network when applying for an opening. Royster (2003) reported that poor and minority youth were less likely than whites to have those kinds of job networks. Smith (2007) found that low-income African-Americans were hesitant to act as network sponsors in case the nominee proved to be a poor worker. Job references are a more bureaucratic version of network sponsorship, involving persons in authority who can attest to a job applicant's skills, or work behavior.

The relevance of this perspective is that students from low income backgrounds are less likely to have family and acquaintance networks that are well-connected in the job world. Lower income students working during college may obtain references and networks through that employment, which they could not easily obtain elsewhere. One implication is that employment during college would be especially important for underprivileged undergraduates when they search for post-college jobs.

A fourth perspective on college employment and post-college earnings raises the possibility that any observed association between working during college and post-college earnings is spurious rather than causal. There might be personality attributes such as ambition, grit, or perseverance (Duckworth, 2018) that predispose individuals to work during college and also lead to superior jobs after college with higher wages, creating a spurious correlation between working in college and higher post-college earnings.

In sum, four mechanisms have been theorized as linking employment during college to higher earnings post college—skill acquisition, signaling, building networks and references, or underlying personality traits. These are not mutually exclusive and our aim in this paper is not to test which of them matters more, a methodological challenge which Bills (2003) suggests is practically impossible. Nor will we claim that college employment is strictly causal, in the sense of eliminating the possibility that “spurious” personality factors underlie both college employment and post-college earnings. As Card (1999, p. 2) argued in an analogous context: “In the absence of experimental evidence, it is very difficult to know whether the higher earnings observed for better-educated workers are *caused* by their higher education, or whether individuals with greater earning capacity have chosen to acquire more schooling.” The same logic applies to the causal status of working in college.

The aim of this paper is more modest: to document the association between working during college and post-college earnings for a large population of undergraduates in relatively unselective public colleges and for several demographic subgroups within that population. The analyses below control statistically for several covariates, and use methods that lessen selection bias in order to provide more conservative estimates of the association between term-time work and post-college earnings. However, these models only address selection on observables, so the possibility of selection on unmeasured characteristics (or of spuriousness) will remain.

## Data and Methods

### Sample

Data are drawn from anonymized records from a large urban multi-campus public university system that merged its students' application and transcript data with state records reporting wage and employment information during and after college. All first-time degree students entering the system between Fall 1999 and Fall 2008 are included in the analyses.

This university system includes community colleges and 4-year colleges. Taken as a whole, the system's student body is emblematic of non-elite, mass higher education. The models presented below separate first-time students who entered 4-year colleges (whom we term “BA attempters”) and those who started in Associates degree programs at community colleges (called “AA attempters”).

The university obtained information from the National Student Clearinghouse (NSC) to identify its students who had transferred outside the system and obtained degrees elsewhere. Degree attainment variables therefore include degrees received from elsewhere as well as degrees completed within the public system.

Complete descriptive statistics are provided in Table A1. Both student sub-populations are young: even in the community colleges traditional-age undergraduates predominate. They are ethnically diverse: in the AA attempter sample, 69% are Black or Hispanic, while in the BA attempter sample, 48% are from these groups. Women constitute 55% of the AA sample and 60% of the BA sample. At the time of entry to college, 61% of the AA attempters and 53% of the BA attempters qualified for Pell grants, indicating a large proportion from families with relatively low income.

Among the AA attempters 58% had no degree by 2014, 31% had earned an Associate degree, and 25% had completed a Baccalaureate degree. Among BA attempters, 29% had no degree, 67% had completed a BA.

### Selection and Limitations of the Analytic Sample

The sample was limited by the availability of post-college wage data: students needed to have non-missing wage data for the outcome period (2013-Q3 through 2014-Q2). Students who were still enrolled in college at the start of the outcome period, 2013-Q3, were omitted. These constraints yielded analytic samples of 103,787 AA attempters and 59,266 BA attempters. Both samples are limited to those who began college and who subsequently remained working in the state. However, administrative data from the university system indicate that 94% of those who earned an associate degree and 83% of those who earned a bachelor degree still reside in the state a decade or more later. The system is in a metropolitan area in which job opportunities are relatively plentiful. For important context, the state under study is one of the largest economies (measured by GDP) in the US, and was not among those states hit hardest by the 2007 recession.

### Dependent Variable

The dependent variable is post-college annual earnings, measured between the third quarter of 2013 and the second quarter of 2014 (the latest data available) as reported by state records. In most analyses, earnings are top-coded at $100,000 per year, to reduce the influence of outliers. Robustness checks also estimated models predicting log earnings and earnings without top coding. Findings were not substantially affected by those alternative specifications.

### Main Independent Variables

Because state administrative records do not indicate work hours or occupation, information on two dimensions of work in college is limited to two dimension: earnings and the duration of paid employment during college, measured in 3-month wage quarters. Optimally, data would contain information on the number of hours worked, the types of work performed, and the sectors in which jobs were located. The models below examine paid employment during the first year of college. Earnings in the first 2 years of college were also examined in robustness checks. Five categories of paid work were constructed and apply both to earnings during the first year of college and to earnings in the year prior to college entry. “No work” means subjects who had zero reported wages in their first year in college; this is used as the reference category for all models. As Table 2 indicates, about 26% of the AA attempters and 33% of the BA attempters did not work for pay during their first year of college.

**Table 2:** Descriptive statistics for students' first-year earnings and 3 year work intensity.

	**AA Attempters**	**BA Attempters**
First year earnings	%	%
Non-worker	26.5	33.0
Low ($0 < x < $5,000)	33.5	37.6
Moderate ($5,000 ≤ x < $15,000)	30.7	26.0
Higher ($15,000 ≤ x < $25,000)	6.4	2.6
Highest (x ≥ $25,000)	2.9	0.9
Prior year earnings		
Non-worker	38.7	47.3
Lower (x < $15,000)	55.3	51.1
Higher (x ≥ $15,000)	6.0	1.6
Semesters of work in first 3 years		
Mean (sd)	7.5 (4.1)	6.7 (4.3)
Sample size (N)	103,787	59,266

The other college employment categories, representing increasing amounts of earnings, are: less than $5,000 per year; $5,000 to $14,999; $15,000 to $24,999, and $25,000 or more. Readers should note that the cut-offs for most of these work categories are quite low: $5,000 per year could be earned by a student working for the minimum wage for under 13 h per week throughout the year, and $15,000 a year is roughly the amount that someone working 40 h a week year-round at a minimum wage job could earn.

A second independent variable captures the duration of paid employment during college and is measured as the number of quarters that a student was employed during the first three years of enrollment in college. In order to avoid conflating earnings during a semester when a student had “stopped out” of college with those resulting from term-time employment, those models that focus on employment duration exclude students who have “stop outs” (semesters when they were not enrolled) during their first 3 years of college.

### Covariates

In the models presented below, the following set of covariates serve as controls:

#### Age

Older students are at a disadvantage in terms of degree completion (Shapiro et al., 2014). This is often attributed to family and work commitments which conflict with schooling. The analytic sample used here is limited to students who entered college aged 18 to 25; age at entry is also included as a covariate in predictive models.

#### Cohort

A set of dummy variables represent the semester and year of college entry from Fall 1999 to Fall 2008 (i.e., cohort fixed-effects). The omitted reference category is the first cohort, Fall 1999. By the 2013/2014 earnings end-point that serves as the dependent variable, students in earlier cohorts had been exposed to more years of work experience on average than more recent cohorts of college entrants. Consequently, one would expect that, *ceteris paribus*, more recent college entrants would have lower post-college earnings than earlier cohorts by that time; i.e., increasingly large negative coefficients for the fixed effects dummies for more recent cohorts. Fall semester entrants also generally fair better than Spring semester entrants, after entering the labor market. These fixed effects may also capture fluctuations in the economic climate over time. Research indicates that timing of entry into the labor market can have important effects on earnings, especially during times of economic recession (Sironi, 2018; Atherwood and Sparks, 2019).

#### Gender

Women have higher rates of college degree completion than men (Buchmann and DiPrete, 2006). Despite this, women are still at a disadvantage in terms of wages; most recent estimates show that women earn about 73 cents on the dollar compared to men of similar educational and occupational attainment. Gender is included as a covariate in all models.

#### Race and Ethnicity

Higher education research has regularly found that Black and Hispanic students have lower rates of degree completion than their White and Asian counterparts (Aud et al., 2010). In these analyses a dichotomous variable indicates whether a student is Black or Hispanic, with White and Asian students as the reference group. The number of native Americans and other race students was very small and those cases were omitted from these analyses.

#### College Major

Students' degree major or last reported major (for non-graduates) is included to account for differences in the labor market value of different fields of study. Carnevale and Cheah (2015) report that both unemployment rates and earnings among employed workers show considerable variation depending on the field of study. Major field of study is recoded into seven categories; the reference category is the business major.

#### Cumulative College Grade Point Average (GPA)

This was measured at graduation or in a student's final semester if a non-graduate.

#### Time Spent in College

This variable is coded as the number of semesters of enrollment between the student's first and last date in the system. In some cases, this includes students who began in a community college and ended in a 4-year college or vice versa. Since this variable is used along with a cumulative credits covariate, the coefficient for time spent in college represents time in college relative to the number of credits earned.

#### Earnings in the Year Before Enrollment

A measure of students' pre-college earnings is included as a proxy for their “human capital” before entering college. We also undertook separate analyses omitting anyone who had earnings prior to entering college.

#### College Credits and Credentials

Transcripts report the cumulative number of undergraduate credits, either at graduation or when the student left the system. If the student graduated with a degree from another institution, they were assigned the typical number of credits of in-system graduates with that degree. Different reference categories for college credits earned are employed in the analyses of BA- and AA-attempters. A set of dummy variables for credits earned and a separate variable for degree attainment was the initial measurement strategy. However, this procedure produced multicollinearity: for example, BA graduates all had 120 or more credits. We therefore constructed a combined variable that reports cumulative credits earned: anyone with over 120 credits in the sample also had a BA degree.

### Analytic Strategy

The first step in the analysis uses logistic regression models to assess association between working during college and the likelihood of students completing degrees (either associates or bachelor's). This step determines whether students in these particular samples evidence the same negative relationship between working during college and graduation identified in prior research. This step has already been demonstrated above in Table 2.

The second step estimates OLS regression models to determine the association between working during college at different levels of earnings during the first year or working for more semesters, and post-college earnings. These are analyzed separately for AA attempters and BA attempters, as the two types of institutions serve different populations, and given that college quality has been demonstrated to have some relationship to student earnings (Andrews et al., 2016). Those regression models are then repeated for various student subsamples such as women, minorities, those who did not work before entering college, and so on, to determine whether the association between term time work and post-college earnings is also evident for each of these subgroups.

Since the data are observational (non-experimental), there may be systematic differences between those who work during college and those who do not, both on measured and unmeasured covariates or “background factors.” If such differences do exist between term time workers and non-workers, OLS regressions could conflate the true association between term time work and later earnings with the effects of those background differences between groups (Heckman, 1979). This is known as selection bias. Adding covariates representing multiple dimensions on which treated and untreated groups might differ does not adequately remove the effects of selection bias (Winship and Morgan, 1999).

Therefore, a third analysis step uses an econometric method for reducing selection bias. First one estimates a “treatment model,” which calculates the probability for each respondent of working (as compared to not working) during college (Guo and Fraser, 2014). The inverse of those probabilities is then used as a weight for each respondent. Inverse probability weighting (IPW) modifies the sample so that the “treated” and “untreated” groups become balanced on measured covariates: they have similar mean values on those variables, removing those background factors as a source of selection bias. A second model, known as the outcome model, then predicts the post-college earnings outcome using a dummy predictor for treatment (work) plus covariates, employing these IPW weights. Selection models of this type are common in medical and economic research but are relatively new to other social sciences. See Xie et al. (2012) for a comprehensive discussion. An example of recent research utilizing selection models in the context of earnings and college can be found in Bockerman's et al. (2019) study of returns to vocational schooling.

These analyses incorporate a recent extension of this general approach known as Augmented Inverse-Probability Weighted regression (AIPW) which enhances robustness and efficiency of estimation (Rubin and van der Laan, 2008; Tan, 2010). AIPW first computes inverse-probability weights predicting treatment status (IPW). Subsequently, separate regressions are estimated for each level of the treatment variable to obtain the treatment-specific outcomes for each. The Average Treatment Effect (ATE) is estimated from the weighted means of each treatment level regression.

One important advantage of the AIPW technique is that if either the treatment model or the outcome model is incorrectly specified that the method nevertheless yields unbiased estimates of treatment, what statisticians call a “doubly robust” measure (Lunceford and Davidian, 2004; Funk et al., 2010; Glynn and Quinn, 2010). These AIPW models provide estimates of the association between term time working and post-college earnings that are less susceptible to selection bias or confounding from measured background factors than those from the OLS models. However, this approach cannot remove the possibility of selection on unmeasured background factors, so the possibility of spuriousness is not eliminated^1^.

## Findings

Descriptive Statistics for our undergraduate sample are provided in Appendix A. Table 3 reports logistic regression models that link undergraduates' working during their first year of college to their likelihood of graduation. For ease of interpretation, Table 3 reports marginal effects in addition to odds ratios. For the BA attempters, the first marginal effect of −0.024 should be read as a BA attempter who worked during the first year of college but earned <$5,000 has a 2.4 percentage point lower chance of graduation with a BA than a student with no work, net of controls. This marginal effect increased to a 6.6 percentage point lower probability of graduating with a BA for students who earned between $5,000 and $14,999 during their first year of college. The marginal effects on graduation are even larger for undergraduates who earned over $15,000 in their first year. At these levels of earnings “employees who study” may be more prevalent.

**Table 3:** Working during college and students' probability of graduation, Logistic Regression—odds ratios, and marginal effects.

	**AA Attempters**		**BA Attempters**	
	**Odds Ratios**	**Marginal Effects**	**Odds Ratios**	**Marginal Effects**
**Earning intensity (ref: non-workers)**				
Low ($0 < x < $5,000)	0.819^***^	−0.047	0.890^***^	−0.024^***^
Moderate ($5,000 ≤ x < $15,000)	0.819^***^	−0.047	0.731^***^	−0.066^***^
Higher ($15,000 ≤ x < $25,000)	0.782^***^	−0.057	0.586^***^	−0.116^***^
Highest (x ≥ $25,000)	0.761^***^	−0.063	0.616^***^	−0.104^***^
Age at entry (years)	0.903^***^	−0.024	0.912^***^	−0.019^***^
Female	1.777^***^	0.133	1.795^***^	0.125^***^
Black/Hispanic	0.615^***^	−0.114	0.455^***^	−0.167^***^
Pell eligible	1.157^***^	0.034	1.019	0.004
Full-time at Entry	1.479^***^	0.088	1.703^***^	0.118^***^
**Prior year earning intensity(ref: Non-worker)**				
Lower (x < $15,000)	0.871^***^	−0.032	0.973	−0.005
Higher (x ≥ $15,000)	0.914^*^	−0.021	0.909	−0.020
Sample size (N)	103,731		59,266	
Pseudo-R^2^	0.051		0.041	

* *p < 0.05*,

*** *p < 0.001*.

*We also control for a student's cohort of entry (not shown in table)*.

Similar negative effects of working upon degree completion are observed for the sample of AA attempters. Students who earned under $5,000 in their first year had graduation rates of 4.7 percentage points lower than non-working students. This grew to 6.3 percent lower among students who earned $25,000 or more.

In sum, for this public university sample there were negative associations between employment during college and graduation, similar to those reported by earlier scholars for other undergraduate samples. However, the following sections will examine a potential benefit of working during college: higher post-college earnings.

Table 4 presents OLS regression models predicting earnings years after college from earnings during the first year of college plus covariates, for those who initially enrolled in an AA program (columns 1 and 2) or a BA program (columns 3 and 4). In each case, the left-hand model contains as predictors only dummies for first year earnings plus demographic variables and entry cohort. The right-hand model adds to those college major, cumulative GPA and credits earned, and time in college, as controls.

**Table 4:** Effects of first-year earnings on post-college earnings in dollars ($). Ordinary least squares regressions.

	**AA attempters**	**AA attempters + educational characteristics**	**BA attempters**	**BA attempters + educational characteristics**
**First year earning intensity (ref: Non-worker)**				
Low ($0 < x < $5,000)	631^***^	1,035^***^	1,270^***^	1,639^***^
Moderate ($5,000 ≤ x < $15,000)	4,252^***^	4,532^***^	3,494^***^	4,332^***^
Higher ($15,000 ≤ x < $25,000)	9,338^***^	9,593^***^	9,208^***^	10,179^***^
Highest (x ≥ $25,000)	18,461^***^	18,155^***^	20,504^***^	20,625^***^
Age at college entry (years)	−475^***^	−495	−655^***^	−756^***^
Female (ref: Male)	−6,664^***^	−7,621^***^	−4,803^***^	−5,908^***^
Black or Hispanic (ref: White or Asian)	−5,173^***^	−3,565^***^	−7,861^***^	−4,135^***^
Pell eligible	−1,408^***^	−1,589^***^	−1,677^***^	−1,648^***^
Full-time at entry	949^***^	300	2,011^***^	763
**Prior year earning intensity (ref: Non-worker)**				
Lower (x < $15,000)	974^***^	1,245^***^	2,030^***^	2,003^***^
Higher (x ≥ $15,000)	4,067^***^	3,747^***^	2,899^**^	2,557^**^
# of semesters enrolled	–	−823^***^	–	−1,308^***^
**Last academic major (ref = Business)**				
STEM	–	130	–	−3,129^***^
Health	–	3,744^***^	–	122
Education	–	−3,311^***^	–	−3,714^***^
Social sciences	–	−2,325^***^		−8,544^***^
Humanities	–	−7,200^***^		−13,179^***^
Liberal arts	–	−682^***^	–	−6,193^***^
Other majors/unknown	–	−890^***^	–	−6,919^***^
**Credits attempted—AA students (ref: 20–59)**				
Less than 20	–	−3,112^***^	–	–
60–89	–	2,342^**^	–	–
90–119	–	4,433^***^	–	–
120 credits or more	–	6,817^***^	–	–
**Credits earned—BA students (ref: <90–119)**				
Less than 20	–	–	–	−6,312^***^
20–59 credits	–	–	–	−5,286^***^
60–89 credits	–	–	–	−2,578^***^
120 credits or more	–	–	–	2,414^***^
Cumulative GPA	–	2,832^***^	–	5,359^***^
Sample Size (N)	103,271	100,596	59,258	58,983
Adjusted R^2^	0.212	0.251	0.232	0.312

*** *p < 0.001*,

** *p < 0.01*.

*We also control for a student's cohort of entry (not shown in table)*.

Column 1 reports that AA attempters who worked during their first year of college earned significantly more after college than students who did not work in college. The post-college benefit increases from $631 per year for those who earned under $5,000 during school, to $4,252 for those who earned between $5,000 and $14,999, to $18,461 for those who earned $25,000 or more during their first year of college.

Column 2 adds covariates such as cumulative credits earned, major, semesters in the university and cumulative GPA. These variables describe later stages of students' careers and therefore represent intervening variables or possible mechanisms impacted by working during one's first year of college that may in turn influence post-college earnings. If these covariates did function this way as intervening mechanisms, then after controlling for these variables the coefficients for working during college would be reduced in magnitude. However, that is not the case: the extended models show large significant associations between work during college and post-college earnings, even after controlling for major, credits earned and degrees, time in college, and cumulative GPA. The post-college earnings premium increases monotonically with higher earnings during the first year of college, but there are substantial “payoffs” to employment during college even for the lower earnings categories.

The covariates are of some interest in their own right: age at entry is significantly and negatively associated with post-college earnings. Women earn less than men, and students from underrepresented minorities earn less than their white and Asian peers. Students had paid work in the year prior to starting college earn more after college than those who did not work for pay. Major or field of study is related to post-college earnings, with Health majors earning significantly more than the reference category (business majors) and education majors earning less.

The coefficients for the set of dummies that represent credits earned by AA attempters provide a useful yardstick against which to compare the benefit of working during college. The reference category for credits is 20 to 59 credits: not enough for an associate degree. Table 4 shows that AA entrants who complete 60–89 credits, enough for the associate degree, have average earnings benefits of $2,342 over the reference category, and those who make it beyond that, presumably by transferring to a BA program, earn even more post-college. By comparison, the earnings boost associated with working in college was $4,532 if one earned between $5,000 and $15,000 in one's first year. In other words, the post-college earnings premium associated with even modest employment during the first year is substantially larger than the earnings bump from completing the number of credits for an Associate degree, confirming Titus (2010) observation in nationally-representative survey data.

Columns 3 and 4 present similar regression models but for BA attempters. Again, both the short model and the longer model show statistically significant higher post-college earnings associated with working during the first year of college: from $1,270 for those who earned under $5,000 in their first year to $20,504 for those who earned $25,000 or more. The longer model that controlled for GPA, major, time in college and credits showed somewhat larger coefficients, suggesting that the post-college benefits of college work are not attributable to those factors.

The other coefficients in the extended model (column 4) are also of interest. The coefficients for age, gender and race/ethnicity are of similar magnitudes as those observed in column 2 among AA attempters. BA attempters who worked prior to beginning college also appear to be at an earnings advantage after college. As for field of study, students studying business in this sample have the highest post-college earnings, with all other categories—including STEM—showing relatively lower earnings.

Readers should also note the coefficients for credits earned in the longer model. The reference category is just below the 120 credits typically needed to graduate with a BA. The coefficient of $2,353 for 120 plus credits can be interpreted as the annual post-college wage benefit associated with completing a BA degree. Again, the post-college earnings boost of $4,232 associated with “moderate” work during the first year of college is larger than that associated with degree completion—$2,414. Table 5 repeats similar regression models for specific subgroups. Only the coefficients for each level of earning in college (in dollars) are reported; the same covariates/controls are used throughout but are not reported in the table due to space constraints (The full models are reported in Tables A2–A7). A post-college earnings premium associated with first-year employment in college is observed for each subpopulation of undergraduates at each level of earnings, suggesting a statistically and substantively significant relationship between employment during college and post college earnings among different kinds of undergraduates. Particularly relevant are the last two rows of Table 5, which show that the positive association between first-year work and post-college earnings is evident even among students who had not worked in the year prior to beginning college.

**Table 5:** Effects of earnings during school on undergraduates' post-college earnings in dollars ($). Ordinary least squares regressions, subgroup analyses.

**Model specifications/filters**	**Low first-year earnings**	**Moderate first-year earnings**	**Higher first-year earnings**	**Highest first-year earnings**	**Model adjusted R-squared**	***N***
AA attempters	1,035^***^	4,532^***^	9,593^***^	18,155^***^	0.251	100,596
AA non-completers	962^***^	4,688^***^	9,910^***^	18,588^***^	0.250	68,417
AA completers	1,174^***^	4,371^***^	9,052^***^	18,081^***^	0.265	32,232
BA attempters	1,639^***^	4,332^***^	10,179^***^	20,625^***^	0.312	58,983
BA non-completers	1,633^***^	4,983^***^	11,199^***^	22,814^***^	0.266	19,617
BA completers	1,719^***^	4,441^***^	9,222^***^	18,868^***^	0.330	39,374
AA attempters, minority students	859^***^	4,559^***^	9,303^***^	17,880^***^	0.240	69,604
BA attempters, minority students	1,462^***^	4,919^***^	10,665^***^	21,274^***^	0.292	28,532
AA attempters, female students	1,149^***^	4,076^***^	9,290^***^	16,584^***^	0.238	55,393
BA attempters, female students	1,144^***^	3,801^***^	9,454^***^	16,175^***^	0.313	35,181
AA attempters, not working before entry	1,335^***^	5,136^***^	13,818^***^	24,874^***^	0.234	39,327
BA attempters, not working before entry	1,853^***^	4,774^***^	10,550^***^	24,314^***^	0.300	27,924

*** *p < 0.001*.

*Full models included in Tables A1–A7*.

Table 6 examines the relationship between duration of employment during college and post-college earnings, controlling for demographic factors, credits earned, college major and so on. There is a consistent pattern that indicates the longer the duration of employment during the first 3 years of college, the larger the associated post-college wage premium. BA attempters who worked for up to 1 year in college earned $2,883 more post-college. Those who worked 1 to 2 years in college earned $4,559 more, and those who worked for 9 or more quarters (2–3 years) earned $6,751 more, on average, post-college. The equivalent associations for AA attempters were $1,258, $3,161, and $6,069.

**Table 6:** Effects of duration of employment on undergraduates' post-college earnings in dollars ($). Ordinary least squares regressions—full models.

	**AA Attempters**	**BA Attempters**
**First 3 years employment continuity (ref: No Employment)**		
Up to 4 quarters	1,258^**^	2,883^***^
5–8 quarters	3,161^***^	4,559^***^
9–12 quarters	6,069^***^	6,751^***^
Age at college entry (years)	−347^***^	−622^***^
Female (ref: Male)	−7,224^***^	−5,945^***^
Black or Hispanic (ref: White or Asian)	−2,206^***^	−3,176^***^
Pell eligible	−1,868^***^	−1,575^***^
Full-time at entry	89	−1,497
**Prior year earning intensity (ref: Non-worker)**		
Lower (x < $15,000)	1,663^***^	2,059^***^
Higher (x ≥ $15,000)	10,006^***^	8,070^***^
# of Semesters enrolled	−1,320^***^	−1,667^***^
**Last academic major (ref = Business)**		
STEM	−78	−2,590^***^
Health	7,357^***^	356
Education	−3,224^***^	−3,318^***^
Social Sciences	−2,184^***^	−9,777^***^
Humanities	−8,274^***^	−14,292^***^
Liberal arts	−962^**^	−6,609^***^
Other majors/Unknown	−1,528^***^	−7,986^***^
**Credits attempted - AA students (ref: 20–59)**		
Less than 20	−3,753^***^	–
60–89	2,697^***^	–
90–119	5,969^***^	–
120 credits or more	6,935^***^	–
**Credits earned—BA Students (ref: <90–119)**		
Less than 20	–	−478
20–59 credits	–	−3,393^***^
60–89 credits	–	−987
120 credits or more	–	381
Cumulative GPA	5,421^***^	7,381^***^
Sample Size (N)	100,596	39,245
Adjusted R^2^	0.283	0.345

*** *p < 0.001*,

** *p < 0.01*.

*We also control for a student's cohort of entry (not shown in table)*.

In sum, for both AA and BA attempters, the duration of employment during the first 3 years of college is associated monotonically with substantially higher wages years later.

### Selection Models

As discussed previously, AIPW treatment models estimate the effect of a treatment on an outcome after correcting statistically for selection bias: differences in measured background characteristics. Table 7 reports average treatment effects (ATE) for BA attempters and for AA attempters. In both cases the treatment is a dichotomy: no paid employment in the first year of college vs. any paid employment. For BA attempters, the average treatment effect was $2,828 per year in post college earnings, while for AA attempters the ATE was $2,962. Both were statistically significant (*p* < 0.001).

**Table 7:** Effects of work during the first year of college on undergraduates' later earnings, Augmented inverse probability weighting (AIPW) treatment effects model.

**Model specifications/Filters**	**Effect of any first year earnings ($)**	**Sample size (N)**
AA Attempters	2,962^***^	100,596
BA Attempters	2,828^***^	59,258

*** *p < 0.001*.

Selection models of any type can only adjust for selection on “observables” or measured covariates. This always leaves open the possibility of selection on “unobservables” or spurious correlation: that there could be some unmeasured factor that was associated both with working in college (the treatment) and with post-college earnings (the outcome).

### Robustness Checks and Threats to Validity

One concern in predictive modeling is that findings might depend upon the particular specification of variables or of the model as a whole and therefore might differ if those specifications were changed. A related concern is whether the observed findings might be driven by outliers or by the inclusion of certain groups. Appendix B presents regression models that use different categories for the variable representing first year earnings and different specifications of the outcome variable such as log earnings after college, or dollar earnings without top-coding. Another robustness check measures earnings during the first 2 years of college, instead of just the first year. Finally, we estimate a model that excluded all the highest earning undergraduates, to see whether they might be driving the observed associations.

In each case, the coefficient for employment during college remains substantial in magnitude and statistically significant. These alternative models, along with the regressions in Table 4 that analyzed specific subgroups, suggest that the strong association of working during college is robust: it does not disappear when models are re-specified and it holds for all the diverse subgroups of undergraduates considered.

## Discussion and Conclusion

Longstanding media stereotypes portray college as a protected interlude between high school and adulthood, a time-out for young adults before life in the real world begins. That portrait may accurately characterize the college experiences undergraduates in past decades, and more privileged undergraduates today. But for the majority of undergraduates nowadays, college life is no time-out, but often a period of competing pressures from commuting, paid work, family obligations, and educational activities (Perna, 2010; Goldrick-Rab, 2016).

Using the traditional student or media stereotype as an ideal, previous researchers understandably focused on paid employment during college as a threat to students' academic performance. Indeed, they were correct in one way: the analyses presented above also show for one state university system that students who work a lot are less likely to graduate. However, prior researchers did not have access to the sort of data we use here; the capacity to merge postsecondary education data with comprehensive employment data at the student level permits researchers to look beyond degree completion outcomes and follow students into their working lives.

Using these newly available data, another more positive side of the story emerges that most previous research overlooked: for students of the multi-campus state university system studied here, working during college clearly pays off in terms of higher earnings in the years after college. This positive economic benefit seems substantial—ranging between approximately $2,000 and $20,000 in the OLS regression models and about $3,000 annually in the models which address selection bias. The positive associations of work during college with post-college earnings also appear widespread and are found for many different kinds of undergraduates. In magnitude, the earnings benefits are as large as those associated with completing the degree.

In the context non-elite mass higher education colleges, such as the system we analyzed, a record of steady work experience may be valued beyond the degree as an additional indicator of dependability and self-discipline that carries weight in distinguishing one mass-college-going job applicant from another. In other words, it suggests a hypothesis that where credential inflation renders degrees less distinctive, “working one's way through college” becomes a useful additional signal. Indeed, the survey cited above suggests that many employers have come to value experience above academic markers when deciding to hire college graduates (cf. Fischer, 2013). Conversely, where credentials retain their distinctiveness (e.g., at the high end of the college prestige pyramid) working during college may be less important for future earnings than the elite college degree itself, which acts as a signal or brand name.

From the perspective of students, working during college not only fulfills an immediate need to earn money, it has also become one more resource—alongside other signals such as pursuing a double major or participating in extra-curricular activities in college, by which to improve their chances of employment in a good job by signaling their exceptional merit to employers. The value of work may also go beyond signals, since prior work experience may impart working students with work relevant “hard” and “soft” skills that allow them to adapt to and succeed in post-college employment. Social networks may also play a role, as students who have spent time at work may indeed acquire contacts, giving them an advantage in the job market. Finally, there is indeed the possibility that those students who worked while attending college were more determined than their peers, and that the same determination shows through in their subsequent earnings. As stated before, the aim here is not to prove or disprove any mechanism by which term-time work leads to higher earnings.

Future research might include audit studies with artificial job applicants to examine whether undergraduates' job histories increase their chances of succeeding with job applications, and whether “working one's way through college” has differential payoffs for graduates depending on the selectivity of the college attended. The data used here were also limited to information about earnings; a more robust model would include information about the number of hours worked, the type of employment, and the industry in which the job(s) were located. Prior research has suggested that more flexible work schedules are more conducive to academic success (Bozick, 2007; Scott-Clayton and Minaya, 2016). It may also be the case that better paying jobs and/or those that match the students' career goals are most strongly correlated with higher post-college earnings. Another limitation is that the data used here do not include information about parental earnings or occupation, both of which are known to be well-correlated with earnings in the next generation. These observable limitations, in addition to any unmeasured differences in student populations, preclude making any causal claims based on our analysis.

The findings we presented above were limited to a single, albeit very large, state university system in an urban area where employment are relatively plentiful; this could limit the generalizability of our findings. They depended upon the availability of data from government sources on earnings and employment before, during and after college. Fortunately, similar data have recently been compiled in several states, and future analyses of these data will help to generalize the findings presented here. Researchers will be able to undertake similar studies for other states and university systems that can review our finding that employment during college is acting as a stepping stone and not only as a stumbling block. At the time of this writing, we are aware of such efforts already underway.

The main implication of this research for educators and policymakers is that one should avoid characterizing undergraduate employment as a threat to academic performance, or as a necessary evil, and instead appreciate that working undergraduates are not only earning much needed income in the short-term but are also enhancing their future long-term earnings prospects. Many colleges already assist their undergraduates in obtaining work in the form of internships, and research suggests that internships are an important factor in hiring (Fischer, 2013). But these are likely to be unpaid and therefore at odds with the increasing financial needs of today's undergraduates. Some colleges—for example Northeastern University—go further and build partnerships with employers whereby undergraduates alternate full-time paid employment with semesters in college, in a sandwich pattern. These partnerships, typically called “co-op” programs, exist at colleges including Georgia Tech, Cornell University and Purdue University, though Northeastern appears unique in enrolling almost all of its students in co-op for at least 1 year (Northeastern University)^2^. Policies like these that perceive undergraduate employment as a positive force and an opportunity for important informal learning and therefore facilitate intertwined employment and study are consistent with the above findings on working during college as a stepping stone for many undergraduates.

## Data Availability Statement

The datasets generated for this study are available on request to the corresponding author.

## Author's Note

A previous version of this manuscript has been released as a report at https://smlr.rutgers.edu/sites/default/files/eerc_working_students_012119.pdf.

## Author Contributions

DD and PA contributed equally to the data analysis and writing.

### Conflict of Interest

The authors declare that the research was conducted in the absence of any commercial or financial relationships that could be construed as a potential conflict of interest.

## Footnotes

^1^Generally, co-op programs seem to be more common in majors in which the undergraduate credential is the terminal degree (e.g., Engineering or Computer Science).

^2^Conceptually there is similarity between the Heckman correction and the AIPW approach, in that both involve a two-stage estimation. But while the focus of Heckman's correction is to address truncated observational data especially for dependent variables, the AIPW method is primarily employed for its robustness to model misspecifications.
