# Supplementary material for: The Relationship Between Work During College and Post College Earnings
Source: Front Sociol. 2019 Dec 10;4:78. doi: 10.3389/fsoc.2019.00078 (PMC8022698; doi:10.3389/fsoc.2019.00078)
Supplement: Supplementary file 1 [file Table_1.DOCX]

TABLE A1

Descriptive Statistics

|  | AA Attempters | BA Attempters |
| --- | --- | --- |
| Mean Earnings in Outcome Period (sd) | 38,814 (23,149) | 46,074 (25,762) |
| Earnings in First Year of College |  |  |
| Non-Worker | 26.5 | 33.0 |
| Low ($0 < x < $5,000) | 33.5 | 37.6 |
| Moderate ($5,000 ≤ x < $15,000) | 30.7 | 26.0 |
| Higher ($15,000 ≤ x < $25,000) | 6.4 | 2.6 |
| Highest (x ≥ $25,000) | 2.9 | 0.9 |
| Earnings in Year Before First Enrollment |  |  |
| Non-Worker | 38.7 | 47.3 |
| Less (x<$15,000) | 55.3 | 51.1 |
| More (x>$15,000) | 6.0 | 1.6 |
| Quarters of Work in First Three Years |  |  |
| Non-Worker | 9.2 | 12.9 |
| 1-4 quarters | 17.2 | 20.7 |
| 5-8 quarters | 24.2 | 23.8 |
| 9-12 quarters | 49.4 | 42.6 |
| Mean Age (sd) | 19.4 (1.7) | 18.5 (1.0) |
| Female | 54.8 | 59.6 |
| Underrepresented Minority | 69.3 | 48.4 |
| Pell Recipient | 61.1 | 53.3 |
| Mean Semesters Enrolled (sd) | 7.2 (4.6) | 9.3 (4.0) |
| Full-time in First Semester | 89.7 | 97.6 |
| Degree Attainment |  |  |
| No Degree Earned | 57.8 | 29.0 |
| AA Earned | 17.3 | 4.5 |
| BA Earned | 25.0 | 66.5 |
| Credits Earned |  |  |
| Less than 20 | 31.7 | 7.8 |
| 20-59 | 19.5 | 11.3 |
| 60-89 | 18.2 | 8.6 |
| 90-119 | 4.2 | 4.5 |
| 120 or more | 26.4 | 67.9 |
| Major Field of Study |  |  |
| Business | 18.2 | 20.5 |
| STEM | 13.2 | 8.6 |
| Health | 10.0 | 6.2 |
| Education | 4.5 | 7.0 |
| Social Sciences | 6.3 | 15.3 |
| Humanities | 4.5 | 7.9 |
| Liberal Arts | 27.8 | 21.5 |
| Other/Unclassified | 15.5 | 13.0 |
| Sample Size (N) | 103,787 | 59,266 |

TABLE A2

Effects of first-year earnings on AA and BA attempters’ post-college earnings in dollars ($), Ordinary Least Squares Regressions (Rows 1 and 4 of TABLE 5)

|  | AA Attempters | BA Attempters |
| --- | --- | --- |
| First Year Earnings Intensity (ref: Non-worker) |  |  |
| Low ($0 < x < $5,000) | 1,035*** | 1,639*** |
| Moderate ($5,000 ≤ x < $15,000) | 4,532*** | 4,332*** |
| Higher ($15,000 ≤ x < $25,000) | 9,593*** | 10,179*** |
| Highest (x ≥ $25,000) | 18,155*** | 20,625*** |
| Age at College Entry (years) | -495 | -756*** |
| Female (ref: Male) | -7,621*** | -5,908*** |
| Black or Hispanic (ref: White or Asian) | -3,565*** | -4,135*** |
| Pell Eligible | -1,589*** | -1,648*** |
| Full-time at Entry | 300 | 763 |
| Prior Year Earnings Intensity (ref: Non-Worker) |  |  |
| Lower (x < $15,000) | 1,245*** | 2,003*** |
| Higher (x ≥ $15,000) | 3,747*** | 2,557** |
| # of Semesters Enrolled | -823*** | -1,308*** |
| Last Academic Major (ref=Business) |  |  |
| STEM | 130 | -3,129*** |
| Health | 3,744*** | 122 |
| Education | -3,311*** | -3,714*** |
| Social Sciences | -2,325*** | -8,544*** |
| Humanities | -7,200*** | -13,179*** |
| Liberal Arts | -682*** | -6,193*** |
| Other Majors/Unknown | -890*** | -6,919*** |
| Credits Earned - AA Students (ref: 20-59) |  |  |
| Less than 20 | -3,112*** | -- |
| 60-89 | 2,342** | -- |
| 90-119 | 4,433*** | -- |
| 120 credits or more | 6,817*** | -- |
| Credits Earned - BA Students (ref: <90-119) |  |  |
| Less than 20 | -- | -6,312*** |
| 20-59 credits | -- | -5,286*** |
| 60-89 credits | -- | -2,578*** |
| 120 credits or more | -- | 2,414*** |
| Cumulative GPA | 2,832*** | 5,359*** |
| Sample Size (N) | 100,596 | 58,983 |
| Adjusted R^2^ | .251 | .312 |

***p<.001 **p<.01 *p<.05

Note: We also control for a student’s cohort of entry (not shown in table)

TABLE A3

Effects of first-year earnings on BA completers and non-completers post-college earnings in dollars ($), Ordinary Least Squares Regressions (Rows 5 and 6 of TABLE 5)

|  | BA Completers | BA Non-Completers |
| --- | --- | --- |
| First Year Earnings Intensity (ref: Non-worker) |  |  |
| Low ($0 < x < $5,000) | 1,708*** | 1,601*** |
| Moderate ($5,000 ≤ x < $15,000) | 4,367*** | 4,856*** |
| Higher ($15,000 ≤ x < $25,000) | 9,142*** | 11,733*** |
| Highest (x ≥ $25,000) | 18,671*** | 22,415*** |
| Age at College Entry (years) | -708*** | -791*** |
| Female (ref: Male) | -4,977*** | -7,910*** |
| Black or Hispanic (ref: White or Asian) | -3,103*** | -4,693*** |
| Pell Eligible | -1,277*** | -2,081*** |
| Full-time at Entry | 599 | 339 |
| Prior Year Earnings Intensity (ref: Non-Worker) |  |  |
| Lower (x < $15,000) | 1,900*** | 2,360*** |
| Higher (x ≥ $15,000) | 1,343 | 3,979** |
| # of Semesters Enrolled | -1,426*** | -886*** |
| Last Academic Major (ref=Business) |  |  |
| STEM | -3,106*** | -689 |
| Health | -42 | 3,651*** |
| Education | -4,656*** | -4,499*** |
| Social Sciences | -10,952*** | 686 |
| Humanities | -15,854*** | -4,139*** |
| Liberal Arts | -8,366*** | -475 |
| Other Majors/Unknown | -9,415*** | 296 |
| Credits Earned (ref: <90-119) |  |  |
| Less than 20 | N/A | -5,229*** |
| 20-59 credits | N/A | -3,037*** |
| 60-89 credits | N/A | -985 |
| 120 credits or more | N/A | -2,448** |
| Cumulative GPA | 7,798*** | 3,129*** |
| Sample Size (N) | 39,374 | 19,617 |
| Adjusted R^2^ | .330 | .266 |

***p<.001 **p<.01 *p<.05

Note: We also control for a student’s cohort of entry (not shown in table)

TABLE A4

Effects of first-year earnings on AA completers and non-completers post-college earnings in dollars ($), Ordinary Least Squares Regressions (Rows 2 and 3 of TABLE 5)

|  | AA Completers | AA Non-Completers |
| --- | --- | --- |
| First Year Earnings Intensity (ref: Non-worker) |  |  |
| Low ($0 < x < $5,000) | 1,332*** | 963*** |
| Moderate ($5,000 ≤ x < $15,000) | 4,435*** | 4,657*** |
| Higher ($15,000 ≤ x < $25,000) | 8,960*** | 9,831*** |
| Highest (x ≥ $25,000) | 17,794*** | 18,341*** |
| Age at College Entry (years) | -260** | -635*** |
| Female (ref: Male) | -5,942*** | -8,223*** |
| Black or Hispanic (ref: White or Asian) | -3,294*** | -3,734*** |
| Pell Eligible | -1,565*** | -1,502*** |
| Full-time at Entry | 1,151** | 60 |
| Prior Year Earnings Intensity (ref: Non-Worker) |  |  |
| Lower (x < $15,000) | 543* | 1,616*** |
| Higher (x ≥ $15,000) | 3,061*** | 4,189** |
| # of Semesters Enrolled | -592*** | -857*** |
| Last Academic Major (ref=Business) |  |  |
| STEM | -21 | 33 |
| Health | 8,104*** | 742* |
| Education | -3,894*** | -2,930*** |
| Social Sciences | -2,414*** | -1,201** |
| Humanities | -7,884*** | -6,560*** |
| Liberal Arts | -1,848** | -698** |
| Other Majors/Unknown | -2,682*** | -213 |
| *Credits Earned (ref: <20-59)* |  |  |
| Less than 20 | N/A | -3,580*** |
| 60-89 credits | N/A | 3,820*** |
| 90-119 credits | N/A | 4,129*** |
| 120 credits or more | N/A | 6,945*** |
| Cumulative GPA | 4,920*** | 2,444*** |
| Sample Size (N) | 32,232 | 68,417 |
| Adjusted R^2^ | .265 | .250 |

***p<.001 **p<.01 *p<.05

Note: We also control for a student’s cohort of entry (not shown in table)

TABLE A5

Effects of first-year earnings on Under-represented minority students’ post-college earnings in dollars ($), Ordinary Least Squares Regressions (Rows 7 and 8 of Table 5)

|  | AA Attempters | BA Attempters |
| --- | --- | --- |
| First Year Earnings Intensity (ref: Non-worker) |  |  |
| Low ($0 < x < $5,000) | 905*** | 1,497*** |
| Moderate ($5,000 ≤ x < $15,000) | 4,569*** | 4,894*** |
| Higher ($15,000 ≤ x < $25,000) | 9,285*** | 10,567*** |
| Highest (x ≥ $25,000) | 17,734*** | 21,126*** |
| Age at College Entry (years) | -433*** | -543*** |
| Female (ref: Male) | -6,595*** | -5,434*** |
| Pell Eligible | -1,210*** | -1,368*** |
| Full-time at Entry | 198 | 11 |
| Prior Year Earnings Intensity (ref: Non-Worker) |  |  |
| Lower (x < $15,000) | 1,046*** | 1,770*** |
| Higher (x ≥ $15,000) | 3,803** | 2,679*** |
| # of Semesters Enrolled | -677*** | -1,016*** |
| Last Academic Major (ref=Business) |  |  |
| STEM | 281 | -269 |
| Health | 2,759*** | 1,632** |
| Education | -2,994*** | 110 |
| Social Sciences | -2,034*** | -5,312*** |
| Humanities | -6,349*** | -8,777*** |
| Liberal Arts | -798*** | -3,148*** |
| Other Majors/Unknown | -868*** | -3,945*** |
| Credits Earned - AA Students (ref: 20-59) |  |  |
| Less than 20 | -2,701*** | -- |
| 60-89 | 2,158*** | -- |
| 90-119 | 3,437*** | -- |
| 120 credits or more | 6,181*** | -- |
| Credits Earned - BA Students (ref: <90-119) |  |  |
| Less than 20 | -- | -4,759*** |
| 20-59 credits | -- | -3,944*** |
| 60-89 credits | -- | -1,807** |
| 120 credits or more | -- | 3,251*** |
| Cumulative GPA | 2,743*** | 4,680*** |
| Sample Size (N) | 69,604 | 28,532 |
| Adjusted R^2^ | .240 | .292 |

***p<.001 **p<.01 *p<.05

Note: We also control for a student’s cohort of entry (not shown in table)

TABLE A6

Effects of first-year earnings on Female students’ post-college earnings in dollars ($), Ordinary Least Squares Regressions (Rows 9 and 10 of Table 5)

|  | AA Attempters | BA Attempters |
| --- | --- | --- |
| *First Year Work Intensity (ref: Non-worker)* |  |  |
| Low ($0 < x < $5,000) | 1,149*** | 1,144*** |
| Moderate ($5,000 ≤ x < $15,000) | 4,076*** | 3,801*** |
| Higher ($15,000 ≤ x < $25,000) | 9,290*** | 9,454*** |
| Highest (x ≥ $25,000) | 16,584*** | 16,175*** |
| Age at College Entry (years) | -349*** | -963*** |
| Female (ref: Male) | -1,732*** | -3,335*** |
| Pell Eligible | -1,281*** | -1,425*** |
| Full-time at Entry | -26 | 1,578* |
| *Prior Year Work Intensity (ref: Non-Worker)* |  |  |
| Lower (x < $15,000) | 906*** | 1,835*** |
| Higher (x ≥ $15,000) | 3,179*** | 3,944*** |
| # of Semesters Enrolled | -639*** | -1,175*** |
| *Last Academic Major (ref=Business)* |  |  |
| STEM | -1,093 | -4,376*** |
| Health | 3,558*** | -403 |
| Education | -4,152*** | -4,572*** |
| Social Sciences | -3,744*** | -9,863*** |
| Humanities | -6,808*** | -13,464*** |
| Liberal Arts | -1,191*** | -6,538*** |
| Other Majors/Unknown | -2,267*** | -8,963*** |
| *Credits Attempted - AA Students (ref: 20-59)* |  |  |
| Less than 20 | -2,226*** | -- |
| 60-89 | 2,301*** | -- |
| 90-119 | 4,752*** | -- |
| 120 credits or more | 8,349*** | -- |
| *Credits Earned - BA Students (ref: <90-119)* |  |  |
| Less than 20 | -- | -5,197*** |
| 20-59 credits | -- | -4,790*** |
| 60-89 credits | -- | -2,635** |
| 120 credits or more | -- | 3,712*** |
| Cumulative GPA | 2,973*** | 5,558*** |
| Sample Size (N) | 55,393 | 35,181 |
| Adjusted R^2^ | .238 | .313 |

***p<.001 **p<.01 *p<.05

Note: We also control for a student’s cohort of entry (not shown in table)

Appendix Table A7: Effects of first-year earnings on non-working at college entry students’ post-college earnings in dollars ($), Ordinary Least Squares Regressions (Rows 11 and 12 of Table 5)

|  | AA Attempters | BA Attempters |
| --- | --- | --- |
| *First Year Work Intensity (ref: Non-worker)* |  |  |
| Low ($0 < x < $5,000) | 1,335*** | 1,853*** |
| Moderate ($5,000 ≤ x < $15,000) | 5,136*** | 4,774*** |
| Higher ($15,000 ≤ x < $25,000) | 13,818*** | 10,550*** |
| Highest (x ≥ $25,000) | 24,874*** | 24,314*** |
| Age at College Entry (years) | 57 | -360* |
| Female | -7,217*** | -5,199*** |
| Black or Hispanic (ref: White or Asian) | -3,052*** | -3,813*** |
| Pell Eligible | -2,015*** | -2,234*** |
| Full-time at Entry | 142 | 672 |
| # of Semesters Enrolled | -897*** | -1,421*** |
| *Last Academic Major (ref=Business)* |  |  |
| STEM | -247 | -2,724*** |
| Health | 5,713*** | 556 |
| Education | -3,158*** | -4,065*** |
| Social Sciences | -1,553** | -8,421*** |
| Humanities | -7,615*** | -13,441*** |
| Liberal Arts | -116 | -6,574*** |
| Other Majors/Unknown | -943** | -7,241*** |
| *Credits Attempted - AA Students (ref: 20-59)* |  |  |
| Less than 20 | -3,099*** | -- |
| 60-89 | 2,566*** | -- |
| 90-119 | 4,495*** | -- |
| 120 credits or more | 7,768*** | -- |
| *Credits Earned - BA Students (ref: <90-119)* |  |  |
| Less than 20 | -- | -6,432*** |
| 20-59 credits | -- | -5,498*** |
| 60-89 credits | -- | -2,668*** |
| 120 credits or more | -- | 2,812*** |
| Cumulative GPA | 3,061*** | 5,462*** |
| Sample Size (N) | 39.327 | 27,924 |
| Adjusted R^2^ | .234 | .300 |

***p<.001 **p<.01 *p<.05

Note: We also control for a student’s cohort of entry (not shown in table)

Appendix Table B1: Effects of first-year earnings on AA and BA attempters post-college earnings in dollars ($), Ordinary Least Squares Regressions – Outcome Variable not Top-Coded at $100,000

|  | AA Attempters | BA Attempters |
| --- | --- | --- |
| *First Year Work Intensity (ref: Non-worker)* |  |  |
| Low ($0 < x < $5,000) | 997*** | 1,444** |
| Moderate ($5,000 ≤ x < $15,000) | 4,650*** | 4,387*** |
| Higher ($15,000 ≤ x < $25,000) | 9,849*** | 11,127*** |
| Highest (x ≥ $25,000) | 20,291*** | 31,106*** |
| Age at College Entry (years) | -513*** | -1,047*** |
| Female (ref: Male) | -8,540*** | -8,763*** |
| Black or Hispanic (ref: White or Asian) | -4,581*** | -5,611*** |
| Pell Eligible | -1,790*** | -2,786*** |
| Full-time at Entry | 298 | 2,704* |
| *Prior Year Work Intensity (ref: Non-Worker)* |  |  |
| Lower (x < $15,000) | 1,297*** | 1,829*** |
| Higher (x ≥ $15,000) | 3,840*** | 3,723* |
| # of Semesters Enrolled | -937*** | -1,687*** |
| *Last Academic Major (ref=Business)* |  |  |
| STEM | -18 | -3,250*** |
| Health | 3,480*** | -2,396** |
| Education | -3,852*** | -6,681*** |
| Social Sciences | -2,494*** | -10,766*** |
| Humanities | -7,848*** | -16,705*** |
| Liberal Arts | -924*** | -8,001*** |
| Other Majors/Unknown | -1,072*** | -9,965*** |
| *Credits Attempted - AA Students (ref: 20-59)* |  |  |
| Less than 20 | -3,547*** | -- |
| 60-89 | 2,421** | -- |
| 90-119 | 4,919*** | -- |
| 120 credits or more | 7,236*** | -- |
| *Credits Earned - BA Students (ref: <90-119)* |  |  |
| Less than 20 | -- | -7,832*** |
| 20-59 credits | -- | -6,963*** |
| 60-89 credits | -- | -4,178*** |
| 120 credits or more | -- | 2,083* |
| Cumulative GPA | 3,087*** | 7,192*** |
| Sample Size (N) | 100,596 | 58,983 |
| Adjusted R^2^ | .224 | .138 |

***p<.001 **p<.01 *p<.05

Note: We also control for a student’s cohort of entry (not shown in table)

Appendix Table B2: Effects of first-year earnings on AA and BA attempters post-college earnings in dollars ($), Ordinary Least Squares Regressions –Natural Log of Outcome

|  | AA Attempters | BA Attempters |
| --- | --- | --- |
| *First Year Work Intensity (ref: Non-worker)* |  |  |
| Low ($0 < x < $5,000) | .041*** | .043** |
| Moderate ($5,000 ≤ x < $15,000) | .158*** | .130*** |
| Higher ($15,000 ≤ x < $25,000) | .316*** | .304*** |
| Highest (x ≥ $25,000) | .517*** | .520*** |
| Age at College Entry (years) | -.015*** | -.021*** |
| Female (ref: Male) | -.191*** | -.130*** |
| Black or Hispanic (ref: White or Asian) | -.064*** | -.078*** |
| Pell Eligible | -.034*** | -.036*** |
| Full-time at Entry | .012 | .036* |
| *Prior Year Work Intensity (ref: Non-Worker)* |  |  |
| Lower (x < $15,000) | .036*** | .052*** |
| Higher (x ≥ $15,000) | .111*** | .098* |
| # of Semesters Enrolled | -.022*** | -.034*** |
| *Last Academic Major (ref=Business)* |  |  |
| STEM | -.016* | -.106*** |
| Health | .064*** | -.054*** |
| Education | -.108*** | -.095*** |
| Social Sciences | -.066*** | -.218*** |
| Humanities | -.231*** | -.357*** |
| Liberal Arts | -.040*** | -.186*** |
| Other Majors/Unknown | -.042*** | -.181*** |
| *Credits Attempted - AA Students (ref: 20-59)* |  |  |
| Less than 20 | -.084*** | -- |
| 60-89 | .051*** | -- |
| 90-119 | .095*** | -- |
| 120 credits or more | .174*** | -- |
| *Credits Earned - BA Students (ref: <90-119)* |  |  |
| Less than 20 | -- | -.142*** |
| 20-59 credits | -- | -.107*** |
| 60-89 credits | -- | -.058*** |
| 120 credits or more | -- | .089*** |
| Cumulative GPA | .072*** | .124*** |
| Sample Size (N) | 100,596 | 58,983 |
| Adjusted R^2^ | .221 | .271 |

***p<.001 **p<.01 *p<.05

Note: We also control for a student’s cohort of entry (not shown in table)

Appendix Table B3: Effects of first-year earnings on AA and BA attempters post-college earnings in dollars ($), Ordinary Least Squares Regressions – Highest Earners Excluded

|  | AA Attempters | BA Attempters |
| --- | --- | --- |
| *First Year Work Intensity (ref: Non-worker)* |  |  |
| Low ($0 < x < $5,000) | 1,011*** | 1,629*** |
| Moderate ($5,000 ≤ x < $15,000) | 4,516*** | 4,333*** |
| Higher ($15,000 ≤ x < $25,000) | 9,600*** | 10,193*** |
| Age at College Entry (years) | -514*** | -788*** |
| Female (ref: Male) | -7,591*** | -5,834*** |
| Black or Hispanic (ref: White or Asian) | -3,528*** | -4,149*** |
| Pell Eligible | -1,607*** | -1,636*** |
| Full-time at Entry | 477* | 1,254*** |
| *Prior Year Work Intensity (ref: Non-Worker)* |  |  |
| Lower (x < $15,000) | 1,264*** | 2,003*** |
| Higher (x ≥ $15,000) | 3,833*** | 3,003** |
| # of Semesters Enrolled | -830*** | -1,314*** |
| *Last Academic Major (ref=Business)* |  |  |
| STEM | 38 | -3,178*** |
| Health | 3,969*** | 128 |
| Education | -3,208*** | -3,837*** |
| Social Sciences | -2,353*** | -8,591*** |
| Humanities | -7,260*** | -13,259*** |
| Liberal Arts | -634** | -6,222*** |
| Other Majors/Unknown | -813*** | -6,978*** |
| *Credits Attempted - AA Students (ref: 20-59)* |  |  |
| Less than 20 | -3,151*** | -- |
| 60-89 | 2,303*** | -- |
| 90-119 | 4,366*** | -- |
| 120 credits or more | 6,825*** | -- |
| *Credits Earned - BA Students (ref: <90-119)* |  |  |
| Less than 20 | -- | -6,531*** |
| 20-59 credits | -- | -5,455*** |
| 60-89 credits | -- | -2,717*** |
| 120 credits or more | -- | 2,339*** |
| Cumulative GPA | 2,860*** | 5,323*** |
| Sample Size (N) | 97,808 | 58,483 |
| Adjusted R^2^ | .213 | .269 |

***p<.001 **p<.01 *p<.05

Note: We also control for a student’s cohort of entry (not shown in table)

Appendix Table B4: Effects of first-year earnings on AA and BA attempters post-college earnings in dollars ($), Ordinary Least Squares Regressions – Using first three years of earnings as main independent variable

|  | AA Attempters | BA Attempters |
| --- | --- | --- |
| *First Year Work Intensity (ref: Non-worker)* |  |  |
| Low (x < $7,500) | 204 | 2,017*** |
| Moderate ($7,500 ≤ x < $20,000) | 2,190*** | 3,789*** |
| Higher ($20,000 ≤ x < $35,000) | 5,086*** | 6,447*** |
| Highest (x ≥ $35,000) | 12,428*** | 12,515*** |
| Age at College Entry (years) | -477*** | -676*** |
| Female (ref: Male) | -7,334*** | -5,921*** |
| Black or Hispanic (ref: White or Asian) | -4,057*** | -4,691*** |
| Pell Eligible | -1,482*** | -1,507*** |
| Full-time at Entry | 43 | 35 |
| *Prior Year Work Intensity (ref: Non-Worker)* |  |  |
| Lower (x < $15,000) | 598*** | 1,410*** |
| Higher (x ≥ $15,000) | 5,658*** | 5,103** |
| # of Semesters Enrolled | -828*** | -1,328*** |
| *Last Academic Major (ref=Business)* |  |  |
| STEM | 313 | -2,592*** |
| Health | 3,976*** | 569 |
| Education | -2,966*** | -3,153*** |
| Social Sciences | -2,260*** | -8,377*** |
| Humanities | -6,738*** | -12,681*** |
| Liberal Arts | -491* | -5,855*** |
| Other Majors/Unknown | -774** | -6,774*** |
| *Credits Attempted - AA Students (ref: 20-59)* |  |  |
| Less than 20 | -3,491*** | -- |
| 60-89 | 2,697*** | -- |
| 90-119 | 5,016*** | -- |
| 120 credits or more | 7,561*** | -- |
| *Credits Earned - BA Students (ref: <90-119)* |  |  |
| Less than 20 | -- | -7,136*** |
| 20-59 credits | -- | -5,873*** |
| 60-89 credits | -- | -2,949*** |
| 120 credits or more | -- | 2,630*** |
| Cumulative GPA | 2,752*** | 5,490*** |
| Sample Size (N) | 100,596 | 58,983 |
| Adjusted R^2^ | .268 | .321 |

***p<.001 **p<.01 *p<.05

Note: We also control for a student’s cohort of entry (not shown in table)
